# Twinning and intertwined microcrystals in an intriguing, yet elusive, mineral

**DOI:** 10.1107/S2052252520014293

**Published:** 2020-10-30

**Authors:** Sven Hovmöller

**Affiliations:** a Department of Materials and Environmental Chemistry, Stockholm University, SE 106 91 Stockholm, Sweden

**Keywords:** electron diffraction, kaliophilite, twinning, intertwined microcrystals

## Abstract

After a hundred years with no solution, the seemingly simple but actually very complex mineral kaliophilite, KAlSiO_4_, is finally revealed by electron crystallography.

Like Sleeping Beauty in the traditional story, after one hundred years of sleep, a number of enigmatic crystal structures are now coming to life. In this issue of **IUCrJ**, the group of Mauro Gemmi in Pisa report the structure of KAlSiO_4_ (Mugnaioli *et al.*, 2020 ▸). This is at least the third complicated crystal structure that has been studied by X-ray crystallography for a century without being solved, waiting for electron crystallography to reach its present level of sophistication. Recently, Ute Kolb’s group in Mainz solved the mineral vaterite (Mugnaioli *et al.*, 2012 ▸) and Ken Inge *et al.* in Xiaodong Zou’s group in Stockholm solved the structure of the much used medicine bis­muth subgallate (Wang *et al.*, 2017 ▸). The reason for this surge in solving hard problems in crystallography is the rapid development of instrumentation, software and theory in electron crystallography.

X-ray crystallography is a (set of) scientific methods for determining atomic structure. Its results are so profound and spectacular, from the first ever correct description of a chemical structure [NaCl by Bragg (1913 ▸)], that one feels obliged to apologize when saying there are cases where other techniques are better. Yet, it is clear from the name of the method, X-ray ‘crystallography’, that it should be applied to crystals. Although very many things around us are crystalline, from NaCl to almost any mineral and including many organic and biological compounds if pure and concentrated, not everything is crystalline. This holds not only for gases and liquids, but also for very many solid objects, from some minerals to biological tissues. Notably, (to the general public) the most spectacular and well known result of X-ray crystallography, the double helix structure of DNA, is strictly speaking not a crystal but a rather well ordered fibre.

Having thus duly given our apologies, we may consider where we may apply variations of the tremendously powerful technique of X-ray crystallography, in order to depict atomic structures in cases where X-ray crystallography fails or at least is limited. The first consideration may be how to overcome the need for (large) crystals. Even a grain of sand, invisible to the human eye, is almost 10 micrometres in each direction and contains some 10^12^–10^15^ atoms. Many minerals are made up of crystals much smaller than this. They may form twins of random or preferred relative orientations or even sets of related but non-identical structures. Luckily there is an alternative to X-rays for such cases; the use of electrons rather than X-rays.

Electrons react orders of magnitude more strongly with matter than do X-rays, and this opens the door to studying smaller crystals and even non-crystalline objects. Unfortunately, this extremely strong interaction has a major drawback: if the sample is more than a few nanometres thick, the electrons may scatter more than once on their way through the sample. This multiple scattering soon leads to uninterpretable results. For many decades, it was believed that this would make electron crystallography unsuitable for solving atomic structures in a fashion similar to X-ray crystallography. Only a very small number of groups pursued structure determination by electron diffraction and electron microscopy. However, in the last decennium, their endurance has proven to be fruitful. A spectacular development in instrumentation, with super-fast and accurate electronic cameras and computer-controlled electron microscopes, has made it possible to collect complete 3D electron diffraction data on a crystal a million times smaller than needed for X-ray crystallography, in but a few seconds. It has turned out that the most severe problem for electron crystallography was not the multiple scattering, but the lack of complete 3D data.

The group led by Mauro Gemmi in Pisa is one those who can take full advantage of the spectacularly improved situation. In collaboration with excellent mineralogists, they are now in a position where many enigmatic mineral structures can be accurately determined by electron crystallography.

Twinning and superstructures, with not only two but numerous intergrowing crystals, are notoriously hard to solve. In a very informative electron micrograph [see Fig. 2 of Mugnaioli *et al.* (2012 ▸)], a typical part of the mineral is shown. Although the sample is less than 100 × 200 nm, it is obvious that there are several small crystals intergrowing. Even without any knowledge in crystallography, it should be clear that it must be very difficult to solve the atomic structure of such a compound. And, for those who know the pros and cons of single crystal and powder X-ray crystallography, it should be evident why this mineral has resisted being solved, in spite of numerous attempts, some even before W. H. Bragg and W. L. Bragg invented X-ray crystallography in 1912. The crystal size is smaller than what is needed even for X-ray powder diffraction.

Now, Gemmi and his group and several experienced mineralogists have at last solved the atomic structure of this mineral. It was possible by electron crystallography. For one thing, the very much stronger interaction of electrons than that of X-rays with matter, makes it possible to use extremely small samples for the crystallographic analysis. It is clear from the above-mentioned figure that although the mineral is heavily twinned, small areas of some tens of unit cells (*i.e.* a few tens of nanometres) may in fact be single crystals.

Electron crystallography has the unique advantage over X-ray and neutron crystallography that both real and reciprocal space can be directly observed. Electron microscopy data, unlike any type of diffraction data, contain the crystallographic structure factor phases.

In addition to the contribution to the development of electron crystallography, the mineral kaliophilite (KAlSiO_4_), studied here, is of general interest for several reasons. It is one of a dozen minerals found with almost the same stoichiometric composition, but with varying degrees of superstructure. The present is by far the most complicated of them all, with a unit cell 27 times larger than the smallest in the series. These compounds are built up by chains and rings of AlO_4_ and SiO_4_ tetrahedra. The rings are mainly 6-rings, but they can vary in how the tetrahedra point upwards (U) or downwards (D). A most intriguing pattern in kaliophilite, making up just one unit cell, is shown in Fig. 1 ▸. The present mineral is the first with three different such schemes. Is this a portal into a world of infinite variations of these (and other!) minerals? It will certainly prompt X-ray crystallographers to keep an eye on what goes on in electron crystallography. In a wide number of fields, also outside mineralogy and pharmaceuticals, the many structures being quickly and accurately solved by electron crystallography should encourage the use of not only single-crystal and powder X-ray and neutron diffraction, but also electron diffraction.

## Figures and Tables

**Figure 1:**
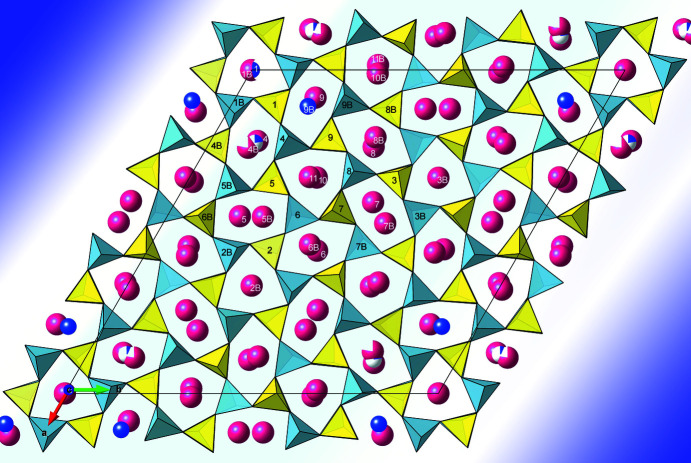
A unit cell of kaliophilite in space group *P*3, *a* = 27.06 and *c* = 8.56 Å;. Si tetrahedra are shown in yellow, Al tetrahedra are shown in sky blue, Na cations are shown in blue and K cations are shown in red. Reproduced from Mugnaioli *et al.* (2020 ▸).
